# Simulation-Based Radiology Training for Medical Technical Assistant Trainees: Quasi-Experimental Study

**DOI:** 10.2196/97489

**Published:** 2026-10-13

**Authors:** Matthias J Witti, Joséphine-Charlotte Coleman, Constanze Richters, Johanna Schönberger, Mary-Anne de Ruijter, Alina Stortz, Melanie Bauer, Raisa Kiriakidou, Matthias Stadler

**Affiliations:** 1 Institute of Medical Education, LMU University Hospital, LMU Medizin, Ludwig-Maximilians-Universität München, Pettenkoferstr. 8a, Munich, 80336, Germany, 089 440053248; 2 medverse GmbH, Munich, Germany; 3 Bayer Medical Care B.V., Radiology, Maastricht, The Netherlands

**Keywords:** simulation-based education, utility value, motivation, radiology training, medical technical assistants

## Abstract

**Background:**

Simulation-based education is increasingly used in medical training to safely develop clinical skills. However, the perceived relevance of a simulation (“utility value”) to a learner’s future work may influence its effectiveness.

**Objective:**

This study evaluated a digital radiology simulation module for medical technical assistant trainees in Germany, examining learning gains, group differences in motivational variables, and the moderating role of utility value.

**Methods:**

Fifty German medical technical assistant trainees (including 24 specializing in radiology and 26 focused on laboratory medicine) completed a pretest and posttest requiring them to arrange 12 steps of a contrast injection procedure in the correct order. All participants then engaged in an interactive computer simulation of the procedure. After the simulation, trainees rated their self-efficacy, as well as the intrinsic value and perceived utility value of the simulation. Learning gains were analyzed using a mixed-design ANOVA on the Spearman footrule distance composite score, and group differences in motivational variables were tested using independent-sample 2-tailed *t* tests. The moderating role of utility value on learning gains was examined using a mixed-design analysis of covariance.

**Results:**

Trainees showed significant improvement in procedural sequencing from pretest to posttest (*F*_1,48_=22.05; *P*<.001; η^2^=0.103), indicating effective learning across both groups. Radiology-specialized trainees reported higher utility value for the training than laboratory-focused trainees (*t*_46_=4.43; *P*<.001; Cohen *d*=1.28), whereas no group differences emerged in intrinsic value or self-efficacy. Contrary to our hypothesis, perceived utility value did not significantly moderate learning gains (*F*_1,46_=0.92; *P*=.34).

**Conclusions:**

The simulation produced robust learning gains regardless of learner specialization or perceived relevance. While utility value perception differed markedly between groups, it did not translate into differential learning outcomes, suggesting that well-designed simulations can be effective across learners with varying levels of task relevance.

## Introduction

Simulation-based learning has become a cornerstone of medical and allied health education, offering learners a safe and controlled environment to practice clinical skills and procedures. Numerous studies have demonstrated that high-fidelity medical simulations can significantly improve the knowledge and procedural skills of learners [1]. Well-designed simulations lead to effective learning by allowing deliberate practice with feedback, and technology-enhanced simulation training has positive effects on learning outcomes in health professions education [2], including improved skill performance and knowledge retention [3,4]. Through virtual scenarios, learners can engage in complex procedures without risk to patients, building competence and confidence for real clinical settings. For example, virtual radiography simulations have enabled learners to practice technical skills (eg, equipment setup and protocol execution) in a safe environment, leading to increased confidence and better preparedness for clinical practice [1]. Medical radiation trainees also appreciate that simulation allows them to practice techniques and skills that may not be possible while on placement due to safety or access limitations [5].

While the general efficacy of simulation-based education is well established, an important question is what factors might further enhance or hinder its impact on learning. One such factor is the perceived relevance or utility value of the simulation to the learner. According to the expectancy-value theory of motivation [6,7], individuals are more motivated and perform better when they expect to succeed and perceive value in a task. Two important components of task value are intrinsic value, which refers to the enjoyment that a learner derives from engaging in a task, and utility value, which reflects the perceived usefulness of a task for one’s future or current goals. Both intrinsic and utility value can strongly promote interest and engagement, yet they do so in different ways: intrinsic value captures the inherent enjoyment and interest experienced during a task, whereas utility value captures the learner’s perception of the task’s relevance to personal or professional goals outside the task itself. Research has shown that learners who perceive high utility value tend to invest more effort, show greater interest, and achieve higher performance [8]. Conversely, if learners view an activity as low in relevance to their goals, their engagement and learning may suffer even if the instructional design is sound. This principle suggests that the same simulation could yield different outcomes depending on the learner’s specialization or career intentions, which shape the utility value they attach to the training.

In the context of medical technical assistant (MTA) education in Germany, trainees typically specialize in distinct fields such as radiology or laboratory medicine during their training. This presents a natural contrast in utility value: a radiology-focused simulation would be directly relevant to trainees specializing in radiological technology, medical technologists for radiology (MTRAs), but might appear less pertinent to those on a laboratory technology track, medical technologists for laboratory analytics (MTLAs). Expectancy-value theory predicts that radiology trainees (high-utility group) may be more engaged and learn more from the simulation than laboratory trainees (low-utility group) even when both groups receive identical training. However, this hypothesis requires empirical examination as other motivational factors and the engaging nature of the simulation itself could also influence the outcome.

This study reports an experimental evaluation of a simulation-based radiology training module for MTA trainees, with a focus on how trainees’ specialization and the associated utility value they derive affect learning and motivational outcomes. The simulation is a digital training module for a computed tomography (CT) contrast injection system (MEDRAD Centargo), developed in collaboration with a medical technology provider (Bayer MEDRAD). The module immerses trainees in the step-by-step process of using the CT injector, from initial setup to performing a contrast-enhanced scan procedure to postprocedure cleanup, organized into 7 sequential chapters reflecting the real-world clinical workflow. By the end of the module, trainees should have a clear mental model of the entire contrast injection workflow and be able to recall the correct sequence of steps involved in using the CT injector safely and efficiently.

Using this simulation as an intervention, the present study addressed 3 main research questions (RQs):

- RQ 1: Does completing the radiology simulation lead to significant learning gains in procedural knowledge from before to after training, and do these gains differ between radiology-specialized and laboratory-specialized trainees?
- RQ 2: Do radiology-specialized trainees report higher intrinsic value, self-efficacy, and utility value of the simulation than laboratory-specialized trainees?
- RQ 3: Does perceived utility value moderate learning gains (ie, do trainees who perceive the training as more useful show larger improvements)?

We hypothesized that, through the simulation activity, trainees in both specializations would improve their sequencing of the steps in the procedure, reflecting effective procedural learning. We further expected that radiology track trainees would rate the simulation as more useful than laboratory track trainees given its direct relevance to radiology practice, whereas we did not formulate directional expectations regarding group differences in intrinsic value or self-efficacy. Finally, based on expectancy-value theory, we anticipated that higher perceived utility value would be associated with greater learning improvement.

## Methods

### Participants and Design

Participants were 50 MTA trainees (second to third trainee year) enrolled in an MTA training program in Germany. All participants had completed basic coursework in their respective tracks but had limited or no prior hands-on experience with the specific radiology procedure featured in the simulation. The sample included 24 trainees specializing in radiology (MTRA track) and 26 specializing in laboratory medicine (MTLA track). This grouping served as a quasi-experimental factor to represent different levels of inherent utility value for the radiology simulation. The majority in both groups were female: MTRA (70%) and MTLA (73%), which is consistent with the typical gender distribution in MTA training programs. Mean age overall was 23.1 (SD 6.93) years, with no significant difference between groups (*P*=.30).

This study used a pretest-posttest design, with all trainees undergoing the same simulation intervention. There was no separate control group as our primary interest was in within-subject learning gains and between-group differences linked to specialization. Trainees first completed a pretest, then the simulation, and immediately afterward a posttest and posttraining questionnaire. Specialization was treated as an independent grouping variable in the analysis. Procedure and testing were identical for both groups, and the study was conducted in a single session per participant.

### Simulation Training System Intervention

The training system was a custom digital simulation module teaching the operation of the MEDRAD Centargo CT power injection system. This system is used in CT radiology suites to administer contrast media and saline during CT scans. The simulation was delivered via a computer, required trainees to perform virtual actions in a clinically realistic sequence, and was structured into 7 chapters corresponding to major phases of the procedure (see Figure 1 for a screenshot of the simulation interface).

**Figure 1.**
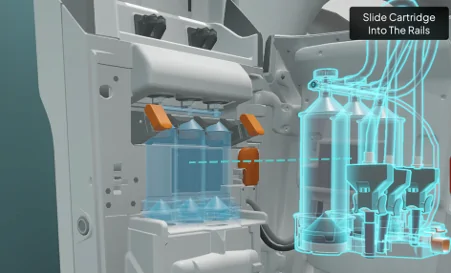
Screenshot of the simulation interface (“Install the daily set”).

### Measures

#### Procedural Knowledge Test (Pretest and Posttest)

The primary learning outcome was the ability to correctly sequence the steps of the radiology procedure following the approach by Bauer et al [9]. The task consisted of a list of 12 key steps involved in the CT injector workflow presented in a randomized order. Trainees were instructed to arrange these steps into the correct chronological order. The pretest assessed baseline knowledge before the simulation; the posttest used the same steps (rerandomized by position) immediately after the simulation to measure learning. For scoring, each participant’s sequence was compared to the expert-established correct order using the Spearman footrule distance, computed as the sum of absolute deviations between each step’s assigned position and its correct position. Lower values indicate greater accuracy.

#### Postsimulation Questionnaire

After the posttest, trainees completed a questionnaire assessing intrinsic value [10], self-efficacy [11], and utility value [10]. Each scale used a Likert-type format from 1 (“strongly disagree”) to 5 (“strongly agree”). The intrinsic value scale (3 items) evaluated enjoyment derived from the simulation. The self-efficacy scale (3 items) measured confidence in performing radiology procedures after training. The utility value scale (3 items) assessed perceived relevance and usefulness of the simulation for the trainee’s future work. Internal consistency (McDonald ω) was high for all 3 scales (intrinsic value: ω=0.95; self-efficacy: ω=0.77; utility value: ω=0.96).

### Procedure

Study sessions were conducted at the MTA training institutes. Trainees were scheduled in regular classes and provided with individual computers and headphones. Two or three researchers were present to ensure standardized administration. Trainees were informed of the general purpose of the session but not told about the specific hypotheses regarding specialization and utility value.

### Data Analysis

We used Jamovi (version 2.3) for all statistical analyses [12]. Following the structure of our 3 RQs, we conducted 3 separate analyses, each using the Spearman footrule distance as the aggregate outcome measure. This composite avoids the dependency inherent in analyzing the 12 individual step positions separately as these constitute a permutation and are therefore not statistically independent. Raw, nontransformed distance scores were used throughout; log-transformation would only be warranted in cases of severe nonnormality.

For RQ 1, we conducted a 2 × 2 mixed-design ANOVA with time (pretest vs posttest) as the within-subject factor and group (radiology vs laboratory) as the between-subject factor. This model tests both the overall learning gain (main effect of time) and whether that gain differed between the 2 trainee groups (time × group interaction).

For RQ 2, we compared the 2 specialization groups on intrinsic value, self-efficacy, and utility value using independent-sample *t* tests. Where the Levene test indicated a violation of the equal variance assumption, the Welch correction was applied.

For RQ 3, we examined whether perceived utility value moderated learning gains using a mixed-design analysis of covariance with time as the within-subject factor and utility value as a continuous covariate. The time × utility value interaction tests whether trainees who rated the simulation as more useful showed larger pre- to posttest improvements.

Effect sizes are reported as η^2^ for ANOVA-based analyses and Cohen *d* for *t* tests. Statistical significance was set at α=.05 for all tests.

### Ethical Considerations

This study was deemed ethically unobjectionable following consultation with the Ethics Committee at the Faculty of Medicine of Ludwig-Maximilians-University Munich (26-0009-KB) and was conducted in accordance with the ethical principles of the Declaration of Helsinki (World Medical Association). Participation was voluntary, and informed consent was obtained.

## Results

### Learning Gains and Group Differences (RQ 1)

Table 1 shows the average position assigned to each procedural step at pretest and posttest alongside the deviation from the correct position. As can be observed, deviations were considerably reduced from pretest to posttest across most steps, most notably for step 9 (“empty or eject the daily set”), where trainees’ placement improved substantially. Figure 2 visualizes this pattern.

**Table 1.** Average step position and deviation from the correct position at pretest and posttest.^a^

Step	Pretest		Posttest	
	Position, mean (SD)	Deviation from correct position	Position, mean (SD)	Deviation from correct position
1. Perform injection	1.74 (2.05)	0.74	1.82 (2.13)	0.82
2. Insert contrast agent and saline containers	2.96 (1.35)	0.96	2.72 (1.84)	0.72
3. Check patient tubing for air bubbles	4.90 (2.32)	1.90	4.26 (2.23)	1.26
4. Prime patient tubing	4.72 (2.09)	0.72	4.50 (2.08)	0.50
5. Dispose of patient tubing	5.68 (1.68)	0.68	5.36 (1.99)	0.36
6. Install patient tubing	7.46 (1.69)	1.46	6.70 (1.74)	0.70
7. Connect patient-side tubing to patient	7.46 (1.18)	0.46	6.82 (1.83)	−0.18
8. Switch on the system	7.80 (1.64)	−0.20	7.94 (1.30)	−0.06
9. Empty or eject the daily set	4.46 (2.67)	−4.54	7.10 (2.81)	−1.90
10. Install the daily set	9.38 (2.10)	−0.62	9.56 (1.64)	−0.44
11. Fill the daily set with fluids	10.86 (2.14)	−0.14	10.44 (2.31)	−0.56
12. Check (test) protocol status	10.58 (2.69)	−1.42	10.78 (2.57)	−1.22

a Deviation=assigned position – correct position; values closer to 0 indicate greater accuracy.

**Figure 2.**
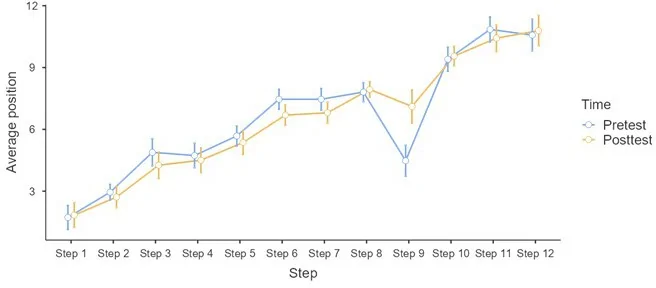
Average position of the 12 procedural steps at pretest and posttest.

Descriptively, the footrule composite score decreased from pretest to posttest in both groups (radiology: mean 16.7, SD 11.5 to 14.3, SD 17.7; laboratory: mean 20.9, SD 11.4 to 13.6, SD 13.7), confirming the direction of the observed main effect of time. The pretest-posttest correlation for the composite score was significant and positive in both groups (radiology: *r*=0.544, 95% CI 0.180-0.777 and *P*=.006; laboratory: *r*=0.417, 95% CI 0.035-0.693 and *P*=.03).

The mixed-design ANOVA showed a significant and medium-sized main effect of time (*F*_1,48_=22.05; *P*<.001; η^2^=0.103), confirming that the simulation effectively improved procedural sequencing accuracy across both groups. The time × group interaction was not significant (*F*_1,48_=2.53; *P*=.12; η^2^=0.012), indicating that learning gains did not differ meaningfully between radiology-specialized and laboratory-specialized trainees. The between-subject main effect of group was also nonsignificant (*F*_1,48_=0.41; *P*=.53; η^2^=0.006), confirming comparable overall performance levels across the 2 tracks. In sum, both groups benefited substantially and equally from the simulation.

### Group Differences in Motivational Variables (RQ 2)

Descriptive statistics for all self-report measures are shown in Table 2.

**Table 2.** Descriptive statistics and intercorrelations for self-report measures.

	Score (1-5), mean (SD)	Intrinsic value	Self-efficacy	Utility value
Intrinsic value	3.79 (1.10)	0.95^a^	0.49^b^	0.59^b^
Self-efficacy	4.07 (0.65)	0.49^b^	0.77^a^	0.42^b^
Utility value	3.48 (1.38)	0.59^b^	0.42^b^	0.96^a^

a Internal consistency (McDonald ω).

b *P*<.001.

Radiology- and laboratory-specialized trainees did not differ significantly in intrinsic value (*t*_45_=1.49; *P*=.14; Cohen *d*=0.44) or self-efficacy (*t*_44_=0.97; *P*=.34; Cohen *d*=0.29). As expected, the groups differed substantially in utility value (*t*_46_=4.43; *P*<.001; Cohen *d*=1.28), with the Levene test indicating unequal variances and the Welch correction applied accordingly. Radiology-specialized trainees rated the simulation as considerably more useful and relevant to their future work (mean 4.29, SD 0.74) than laboratory-specialized trainees (mean 2.79, SD 1.43). The large effect size confirms the effectiveness of the specialization grouping in creating differential utility perceptions.

### Utility Value as a Moderator of Learning Gains (RQ 3)

The mixed-design analysis of covariance with utility value as a continuous covariate confirmed the overall learning gain with a significant main effect of time (*F*_1,46_=6.79; *P*=.01; η^2^=0.035). However, the time × utility value interaction was not significant (*F*_1,46_=0.92; *P*=.34; η^2^=0.005), indicating that perceived utility value did not moderate the degree of learning improvement. The between-subject effect of utility value was similarly nonsignificant (*F*_1,46_=0.32; *P*=.58; η^2^=0.005). Trainees who found the simulation more relevant did not show larger learning gains than those who found it less relevant. Descriptively, the direction of the covariate effect was positive. Trainees with higher utility value scores showed marginally larger pre- to posttest gains (*r*=0.096, 95% CI –0.194 to 0.370; *P*=.52), consistent with, though far weaker than, theoretical expectations. Raw, unadjusted bivariate correlations between utility value and the footrule composite score were similarly weak and nonsignificant at both pretest (*r*=0.085, 95% CI –0.204 to 0.361; *P*=.57) and posttest (*r*=–0.024, 95% CI −0.306 to 0.262; *P*=.87), confirming that utility value was not meaningfully associated with performance at either time point.

## Discussion

### Principal Findings

This study investigated the effectiveness of a simulation-based radiology training module for MTA trainees and examined how perceived utility value influenced learning outcomes. The results demonstrated that the simulation was an effective learning tool: trainees significantly improved their knowledge of the procedural sequence from pretest to posttest, confirming that even a single simulation session can yield meaningful procedural knowledge gains. Importantly, these gains were consistent across both radiology-specialized and laboratory-specialized trainees, suggesting that the simulation was equally effective regardless of specialization background. Contrary to our hypothesis, however, perceived utility value did not significantly moderate learning gains. Trainees who found the simulation more relevant to their future work did not show measurably larger improvements than those who perceived it as less relevant.

### Theoretical Implications: Expectancy Value in Simulation Learning

Our findings offer a partial, rather than full endorsement of expectancy-value theory in this simulation context. The group difference in utility value was large and consistent with theoretical predictions: radiology-specialized trainees rated the simulation as substantially more relevant than their laboratory-specialized peers. However, this difference in perceived relevance did not translate into differential learning outcomes either at the group level or when utility value was modeled as a continuous predictor of individual learning gains. This null moderation finding suggests that utility value, while a meaningful dimension of task motivation, does not uniformly amplify learning in simulation-based contexts, particularly when the simulation itself is well designed, engaging, and instructionally scaffolded.

One possible explanation is that the simulation’s interactive design and immediate feedback structure created sufficient conditions for effective procedural learning across all trainees, effectively compensating for lower utility perceptions in the laboratory group. When the learning environment is highly structured and cognitively supportive, motivational differences between learners may matter less than in less scaffolded settings [3,4]. Alternatively, the sequencing task may have been learnable by most trainees regardless of motivation, and utility value effects might emerge more clearly in longer or more cognitively demanding interventions. Future research using more complex procedural tasks or longer training durations may be better equipped to detect motivational moderation effects. Additionally, immediate posttests may not fully capture the influence of utility value as its effects may be more apparent over time than after a single training session. If utility value promotes deeper engagement or more effortful processing during learning, its benefits may only become apparent once the initial performance gains from immediate feedback have faded. Therefore, delayed retention measures may offer a more sensitive test of motivational moderation than performance assessed directly after training.

Interestingly, intrinsic value and self-efficacy were uniformly high across both groups and did not differentiate learning outcomes. The simulation appeared to capture trainees’ interest and confidence regardless of their specialization, suggesting that well-crafted interactive simulations can engage diverse learner populations. Authors and journals alike have a responsibility to transparently report null findings to prevent publication bias, and the null moderation result reported in this study contributes important empirical evidence on the boundary conditions of expectancy-value theory in simulation-based medical education.

### Practical Implications: Designing and Targeting Simulations for Maximum Impact

From a practical standpoint, our findings offer nuanced guidance for educators and simulation designers. First, the consistent learning gains across both groups suggest that high-quality, feedback-rich simulations can be effective equalizers, supporting procedural knowledge acquisition even among learners who perceive the content as less directly relevant to their career. For heterogeneous cohorts, this is encouraging: simulation-based training need not be restricted to those with the most obvious career alignment.

Second, while utility value did not moderate learning outcomes in this study, the marked group difference in utility perceptions (Cohen *d*=1.28) is itself noteworthy. In longer interventions, more sustained training programs, or contexts in which motivation may play a greater role over time, utility value could yet prove consequential. Framing interventions that highlight the broader relevance of procedural skills, such as structured reflection exercises connecting the simulation to trainees’ future professional roles [13], remain a promising avenue for instructional design even if their effect on short-term learning gains was not confirmed in this study.

Third, the uniformly high intrinsic value and self-efficacy scores attest to the simulation’s success in creating an engaging and confidence-building learning experience. Features likely contributing to this include interactive hands-on tasks, immediate feedback, and clinical authenticity. These qualities align with established principles of effective simulation design [4,14,15] and should continue to guide the development of training modules across specializations.

### Limitations

Several limitations warrant consideration. The sample consisted exclusively of German MTA trainees, limiting generalizability to other educational systems or more advanced learner populations. Specialization was not randomly assigned, introducing potential confounds between group membership and other learner characteristics. The study assessed only short-term procedural learning; longer-term retention and clinical transfer remain beyond its scope. The outcome measure, a sequencing task, captures procedural knowledge but not complex decision-making or interpersonal skills, where motivational moderation might operate differently.

Finally, the self-report scales, while adapted from established instruments, were short (3 items each) and administered only once after training, which may have limited sensitivity to within-person motivational variation. Future studies could complement self-reports with behavioral engagement measures such as time on task or exploration frequency within the simulation to capture motivation more dynamically.

### Conclusions

This study demonstrates that simulation-based training effectively supports the acquisition of complex procedural knowledge among MTA trainees. The simulation produced robust and equivalent learning gains across radiology- and laboratory-specialized trainees, indicating that well-designed interactive simulations can be effective regardless of learners’ perceived task relevance. While utility value differed substantially between groups, as expected, it did not moderate individual learning gains, providing important evidence on the boundary conditions of expectancy-value theory in simulation-based medical education. Future research should examine whether motivational moderation emerges in more complex, longer, or retention-focused learning scenarios and should consider experimental manipulation of utility value to test its causal role more directly.

## Abbreviations

CT: computed tomography
MTA: medical technical assistant
MTLA: medical technologist for laboratory analytics
MTRA: medical technologist for radiology
RQ: research question
